# A  Phase IV Study of the Safety and Efficacy of CinnoPar^®^ in Iranian Patients with Osteoporosis

**DOI:** 10.1155/2021/7584308

**Published:** 2021-05-31

**Authors:** Ahmadreza Jamshidi, Farhad Gharibdoost, Sima Sedighi, Asghar Hajiabbasi, Amir-Hossein Salari, Alireza Khabbazi, Peyman Mottaghi, Ahmad Tahammoli Roudsari, Mehrdad Aghaei, Irandokht Shenavar Masooleh, Araz Sabzvari, Nassim Anjidani

**Affiliations:** ^1^Rheumatology Research Center, Tehran University of Medical Sciences, Tehran, Iran; ^2^Bones, Joints, and Connective Tissue Research Center, Golestan University of Medical Sciences, Gorgan, Iran; ^3^Guilan Rheumatology Research Center, Department of Rheumatology, Razi Hospital, School of Medicine, Guilan University of Medical Sciences, Rasht, Iran; ^4^Connective Tissue Diseases Research Center, Tabriz University of Medical Sciences, Tabriz, Iran; ^5^Department of Internal Medicine, Isfahan University of Medical Sciences, Isfahan, Iran; ^6^Department of Internal Medicine, School of Medicine, Hamadan University of Medical Sciences, Hamadan, Iran; ^7^Golestan Rheumatology Research Center, Golestan University of Medical Sciences, Gorgan, Iran; ^8^CinnaGen Medical Biotechnology Research Center, Alborz University of Medical Sciences, Karaj, Iran; ^9^Orchid Pharmed Company, Tehran, Iran; ^10^Medical Department, Orchid Pharmed Company, Tehran, Iran

## Abstract

The safety of teriparatide has been studied in various phase III and phase IV trials. However, a postmarketing study of the biosimilar of teriparatide, CinnoPar^®^, has not been conducted on Iranian patients. This was a phase IV study conducted on osteoporotic patients who received an Iranian teriparatide biosimilar with a dose of 20 *μ*g daily. The primary outcome of this study was to monitor for adverse events (AEs). Effectiveness as the secondary outcome was measured using the EQ-5D quality-of-life questionnaire and back pain Visual Analogue Scale (VAS) score. Among 193 analyzed patients between September 2015 and March 2019, the most common AEs were hypercalcemia (4%), nausea, and pain (3%). No deaths, serious AEs, or other significant AEs occurred in this study. The mean EQ-5D scores decreased after the course of the treatment from 2.3 ± 0.66 at the baseline to 2 ± 0.66. The mean back pain VAS scores also decreased from 4.9 ± 3.6 at baseline to 1.8 ± 2.1 at the end of the study. Both changes were statistically significant (*p* < 0.001). Consistent with the findings of previous studies and the drug monograph, no new safety concern was observed with this biosimilar teriparatide, and the drug was effective based on the VAS score and EQ-5D in osteoporotic patients.

## 1. Introduction

Osteoporosis is defined as decreased bone mineral density (BMD), which results in increased fracture risk and its subsequent complications [1]. In the United States, the prevalence of osteoporosis at the femoral neck or lumbar spine is estimated to be 10% in adults older than 50 years of age [2]. In Iran, it is estimated that 17% of the adults over 30 years of age have osteoporosis in the lumbar spine and 35% have osteopenia [3]. The high prevalence of this disease in Iran may also increase the risk of its complications.

Osteoporosis, thus, imposes a significant financial burden on the patients, their caregivers, and healthcare systems and also decreases patients' quality of life and increases their morbidity and mortality [4]. Pharmacological management consists of antiresorptive drugs such as bisphosphonates and anabolic options such as teriparatide, a recombinant human parathyroid hormone [5]. It works in a similar fashion to endogenous PTH by increasing bone turnover [6].

There are a few teriparatide biosimilar products across the world [7, 8]. As defined by the European Medicines Agency (EMA), a biosimilar is a biological product highly similar to a reference biological product with no clinically significant difference between the two in terms of efficacy and safety [9]. The lower cost of biosimilars reduces the financial burden on healthcare systems, which is particularly helpful in developing countries such as Iran. The development of biosimilars will in turn increase patients' access to vital biologic drugs and will also drive innovation by the competitor companies [10]. CinnoPar^®^ is an Iranian biosimilar teriparatide produced by CinnaGen biopharmaceutical company. This product was compared with the comparator Forteo® in terms of efficacy and safety in a previous phase III clinical trial [11]. In this phase IV study, we aimed to evaluate the safety and effectiveness of CinnoPar^®^ in osteoporotic patients.

## 2. Methods

### 2.1. Design

The present trial was a phase IV, observational, cohort, single-arm, prospective study conducted in multiple centers across seven cities of Iran from September 2015 to March 2019 to evaluate the safety and effectiveness of Iranian biosimilar teriparatide in patients diagnosed with osteoporosis. Patients were followed up for 12 months after the start of the study. This trial was conducted in accordance with the Good Clinical Practice (GCP) and ethical principles. Patients were informed of their participation in a clinical trial.

### 2.2. Patient Characteristics

The inclusion criterion was osteoporotic patients diagnosed with BMD, and there were no exclusion criteria for this study. Normal status was defined as T-score ≥−1.0, osteopenia as −2.5< T-score <−1.0, osteoporosis as T-score ≤−2.5, and severe osteoporosis as T-score ≤−3.5 or ≤−2.5 with a fragility fracture. The patient's characteristics were recorded by a physician in a booklet at the baseline visit. Four booklets were allocated to each patient, and each booklet covered three months of drug administration. At the first visit, the recorded characteristics comprised demographic data, habitual history, and past medical history, including allergic history, both renal and hepatic impairments, and other medical conditions. If available, the exact diagnosis of each patient's medical condition together with the diagnosis date, last BMD results, and pertinent laboratory data were recorded in this visit. Previous medications that each patient had received for osteoporosis treatment, previous fractures due to osteoporosis, and back pain experience due to spinal fracture were written as well. On the subsequent visits, physicians recorded each patient's course of the disease, adverse events (AEs), and patient's concomitant medications. EQ-5D questionnaires for health quality assessment in the booklets were filled at the baseline and 12^th^ month. Back pain was evaluated using a visual analogue scale (VAS) score at baseline, 3^rd^, 6^th^, 9^th^, and 12^th^ months.

### 2.3. Treatment

The patients received daily subcutaneous injections of teriparatide (CinnoPar^®^) 20 *μ*g provided as a pen or vial. During the study, the dosage form changed from vial to pen for its convenience and decreased usage error. Patients were suggested to inject an Iranian biosimilar of teriparatide at night before sleep to prevent orthostatic hypotension. Calcium and vitamin D supplements were advised in all of the patients unless there was a precaution.

### 2.4. Outcomes

The primary outcome was to monitor the adverse events throughout the study. Causality assessment of the AEs was performed according to the WHO criteria [12]. Hypercalcemia, as the most common AE reported for teriparatide, was defined as serum calcium >10.5 mg/dL in this study. The secondary outcome was the evaluation of quality of life using EQ-5D and back pain VAS score in those experiencing such pain. The lower EQ-5D score represents a better quality of life.

### 2.5. Statistical Analysis

Descriptive analysis of baseline characteristics was reported using mean ± SD for continuous variables and frequency (percentage) for categorical variables. For each AE, data were summarized using incidence according to system organ class and preferred term of AEs. In addition, causality assessment was performed, and its results were reported by frequency and percentage. A generalized estimating equation (GEE) model was used in order to analyze the patients' back pain VAS score. The trend of improvement with regard to the EQ-5D, from baseline to the last visit, was assessed by the paired *t*-test. The EQ-5D score was calculated for each patient by the average score of five questions.

## 3. Results

### 3.1. Demographic Information

A total of 193 patients were included and analyzed in this study. The majority of the patients were females with a mean ± SD age of 65 ± 11 years. Demographic data and baseline characteristics are presented in Table 1. Most of the cases were diagnosed to have spinal osteoporosis, representing 81% of the patients. According to the T-score of the hip at baseline, 48 (53%) out of the 90 osteoporotic patients had severe osteoporosis. Based on the T-score of the spine at baseline, 96 (75%) out of the 128 osteoporotic patients had severe osteoporosis. Thirty-seven patients discontinued the therapy. The most common reason for discontinuation was patient preference (21 cases). The mean ± SD serum calcium of the patients at baseline and months 3, 6, 9, and 12 was 9.4 ± 0.55, 9.4 ± 0.60, 9.4 ± 0.62, 9.3 ± 0.46, and 9.3 ± 0.45 mg/dL, respectively. The mean ± SD of serum vitamin 25-hydroxyvitamin D level at baseline and 12 months was 36.3 ± 21.9 and 35 ± 10.4 ng/mL, respectively.

### 3.2. Safety

The incidence of AEs among the 193 included patients is listed in Table 2. Hypercalcemia was the most commonly observed side effect of CinnoPar^®^ (4%).

Ninety-five percent of the observed AEs were considered at least possibly related based on the causality assessment of the AEs. All observed AEs were of grade 1 or 2, and no deaths, serious AEs, or other significant AEs occurred in this postmarketing study.

### 3.3. Efficacy

**Figure 1:**
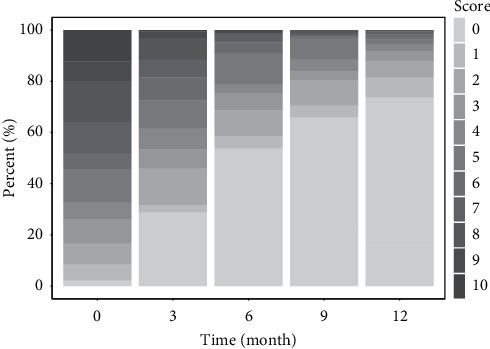
Back pain VAS score during the study. The darker the color, the higher the mean back pain VAS score.

The number of patients with back pain decreased during therapy. At baseline, 78% of the patients had back pain, and this number decreased to 61% at the 12^th^ month. At baseline, 151 patients (78%) had back pain, whereas 44 patients (61%) had back pain at the end of the study. The mean back pain VAS score of the patients at baseline and the end of the study was 4.9 ± 3.6 and 1.8 ± 2.1, respectively. The mean back pain VAS score significantly changed during the study (*p* < 0.001).  Figure 1 depicts the trend of change in the back pain VAS score during the study period.

The mean EQ-5D score before and after therapy significantly improved (*p* < 0.001). The corresponding scores were 2.3 ± 0.66 and 2 ± 0.66, respectively, with a mean (95% CI) difference being −0.3 (−0.43 to −0.17).

## 4. Discussion

In this postmarketing study, twenty-one patients experienced at least one adverse event, accounting for 11% of the patients. The observed AEs were among the common AEs reported for the teriparatide treatment. In the causality assessment of the adverse events, 95% were deemed at least possibly related to teriparatide. It should be noted that we could not perform rechallenge and dechallenge of the treatment, which hindered a more precise causality assessment. In the 24-month Japan Fracture Observational Study (JFOS), 1996, patients who initiated 20 *μ*g daily teriparatide were recruited and 15% of the patients had at least one adverse event [13].

In our study, the frequency of hypercalcemia, the most common AE, was 4%. The landmark phase III clinical trial of teriparatide on postmenopausal women with osteoporosis reported 11% for mild hypercalcemia incidence in the 20 *μ*g group [14]. The higher incidence could be due to the fact that the patient population was strictly postmenopausal women, in whom hyperparathyroidism and hypercalcemia are more common [15]. Other studies mentioned comparable frequency for this AE. For instance, in Hadji et al.'s phase III study comparing the efficacy of 20 *μ*g teriparatide daily with risedronate for 18 months in postmenopausal women, 4% of patients in the teriparatide group had hypercalcemia [16]. In another phase III study by Saag et al. comparing teriparatide with alendronate in 214 osteoporotic patients receiving 20 *μ*g teriparatide daily for 18 months, the incidence of hypercalcemia was 4% [17]. Obermayer‐Pietsch et al. studied the effects of 20 *μ*g daily teriparatide for 24 months in 503 postmenopausal women with severe osteoporosis, and hypercalcemia frequency was reported to be 5% [18].

We reported an incidence of 3% for nausea. In the phase IV study by Kendler et al. comparing the efficacy of 20 *μ*g teriparatide with risedronate in postmenopausal women at high risk for fracture for 24 months, nausea incidence was 5%, which was consistent with our results [19]. Similarly, in the Extended Forsteo Observational Study (ExFOS) conducted for 24 months on 1611 patients with severe osteoporosis, the incidence of 4% was for this AE.

Table 3 presents the frequency comparison of other AEs with studies that mentioned those AEs with the same term as ours. This table demonstrates the relatively similar incidence of AEs between our study and international reports, which administered 20 *μ*g teriparatide.

We also found no cases of osteosarcoma. As mentioned, there were no deaths or serious AEs. However, there were two AEs that led to drug discontinuation; one was a case of headache, and the other was a case of injection site pain.

The mean back pain VAS score of patients decreased by 64%, which was statistically significant and showed the efficacy of Iranian teriparatide biosimilar in decreasing pain (*p* < 0.001). Other studies showed a significant improvement in this domain as well. In line with our findings, in Langdahl et al.'s study, the back pain VAS score had a significant change of 46% (*p* < 0.0001) after 24 months [20]. Similarly, Songpatanasilp et al. reported a decrease of 53% in the back pain VAS score after 12 months [21]. Both aforementioned studies suggest that back pain reduction may be associated with the reduction in vertebral fractures.

The EQ-5D score showed a significant decrease of 13% (*p* < 0.001), suggesting an improvement in patients' quality of life during the study period. In comparison, the reported scores in the study of Ljunggren et al. conducted on 1581 patients for 18 months can be expressed as a 24% improvement [22]. Finally, the study of Songpatanasilp et al. represented a 23% improvement in the mean EQ-5D health state after 12 months. According to these results, teriparatide is effective in decreasing back pain VAS score and improving the quality of life.

Our study had several limitations. Our sample size could be larger to detect rare AEs, and the study duration could be longer to cover the maximum approved duration of usage for teriparatide, which is 24 months, to provide more accurate findings to correspond with the real-world usage. Patients drop out from the study was another limitation. Not all patients were followed up for a full 12 months, and this was predictable due to the observational nature of this study. In addition, we did not evaluate the safety of CinnoPar^®^ in off-label indications. Future postmarketing studies can focus on such indications.

## 5. Conclusions

In this phase IV, observational, cohort study, a total of 193 osteoporotic patients were included and received 20 *μ*g teriparatide (CinnoPar^®^) subcutaneous injections daily based on physicians' routine clinical practice. The present study shows a favorable safety profile for CinnoPar^®^ in osteoporotic patients.

## Figures and Tables

**Table 1:** Patients' demographic and baseline characteristics.

Variable	Result
Population	193
Age (y)	65 ± 11
Height (cm)	156 ± 8.7
Weight (kg)	64 ± 12.2
BMI	26.1 ± 4.8
Female	178 (92)
Smoking	8 (4)
Alcohol consumption	7 (4)
Tea/coffee consumption ≥1 cup per day	167 (87)
Prior osteoporosis treatment	
Total	93 (48)
Calcitonin	2 (2)
Bisphosphonate	91 (98)

Medical history	
Allergic diseases	15 (8)
Hepatic impairment	4 (2)
Renal impairment	10 (5)
Gastrointestinal diseases	20 (10)
Rheumatic diseases	48 (25)
Thyroid dysfunction	13 (7)
Parathyroid dysfunction	1 (1)

Osteopenia/osteoporosis diagnosis	
As per T-score of the hip	
Screened	160 (83)
Normal	7 (4)
Osteopenia	63 (39)
Osteoporosis	90 (56)
As per T-score of the spine	
Screened	158 (82)
Normal	7 (4)
Osteopenia	23 (15)
Osteoporosis	128 (81)

Total serum calcium (mg/dL)	
Baseline	9.4 ± 0.55

Data are presented as mean ± SD or *n* (%), where applicable. SD = standard deviation.

**Table 2:** Incidence of adverse events by system organ class.

System organ class	*N* (%)	Preferred term name	*N* (%)
Cardiac disorders	1 (1)	Tachycardia	1 (1)
Endocrine disorders	7 (4)	Hypercalcemia	7 (4)
Gastrointestinal disorders	7 (4)	Nausea	6 (3)
		Vomiting	1 (1)
		Dyspepsia	2 (1)
General disorders and administration site conditions	7 (4)	Pain	6 (3)
		Asthenia	1 (1)
		Injection site pain	1 (1)
Musculoskeletal and connective tissue disorders	3 (2)	Muscle spasms	2 (1)
		Spinal fracture	1 (1)
Nervous system disorders	5 (3)	Headache	2 (1)
		Insomnia	2 (1)
		Seizure	1 (1)
		Dizziness	2 (1)
Respiratory, thoracic, and mediastinal disorders	2 (1)	Dyspnoea	2 (1)
Skin and subcutaneous tissue disorders	2 (1)	Rash	2 (1)
Vascular disorders	1 (1)	Hypotension	1 (1)
Patients with at least 1 AE, total			21 (11)

**Table 3:** Comparison of some of the AEs with other studies (in percent).

	Our study	Neer et al.	Kendler	Langdahl et al.	Saag et al.	Hadji et al.	Obermayer‐Pietsch et al.
Hypercalcemia	4	11			4	4	5
Nausea	3		5	4			
Headache	1			3	8		7
Dizziness	1	9	4		7		5
Pain	3		2				
Rash	1				1		
Insomnia	1				5		
Muscle spasms	1				4	9	
Dyspepsia	1				3		3
Vomiting	1					3	
Spinal fracture	1					1	

## Data Availability

The data that support the findings of this study are available from the corresponding author. These data are confidential and are held in a local database, not available publicly.
